# Obesity drives adipose-derived stem cells into a senescent and dysfunctional phenotype associated with P38MAPK/NF-KB axis

**DOI:** 10.1186/s12979-023-00378-0

**Published:** 2023-10-11

**Authors:** L K Grun, R M Maurmann, J N Scholl, M E Fogaça, C R R Schmitz, C K Dias, J Gasparotto, A V Padoin, C C Mottin, F Klamt, F Figueiró, M H Jones, E C Filippi-Chiela, F C R Guma, F M Barbé-Tuana

**Affiliations:** 1 Graduate Program in Pediatrics and Child Health, School of Medicine, Pontifical Catholic University at Rio Grande do Sul (PUCRS), Porto Alegre, Brazil; 2 Graduate Program in Cellular and Molecular Biology, School of Health, Sciences, and Life, Pontifical Catholic University at Rio Grande do Sul (PUCRS), Porto Alegre, Brazil; 3 Group of Inflammation and Cellular Senescence, Immunobiology Laboratory, School of Health Sciences and Life, Pontifical Catholic University at Rio Grande do Sul (PUCRS), Porto Alegre, Brazil; 4 Graduate Program in Biological Sciences: Biochemistry, Federal University at Rio Grande do Sul (UFRGS), Porto Alegre, Brazil; 5 Institute of Biomedical Sciences, Federal University at Alfenas, Alfenas, Brazil; 6 Graduate Program in Medicine and Health Sciences, Pontifical Catholic University at Rio Grande do Sul (PUCRS), Porto Alegre, Brazil; 7 Institute of Basic Health Sciences, Department of Morphological Sciences, Federal University at Rio Grande do Sul (UFRGS), Porto Alegre, Brazil; 8 Experimental Research Center, Hospital de Clínicas de Porto Alegre, Porto Alegre, Brazil; 9 Center for Biotechnology, Federal University at Rio Grande do Sul (UFRGS), Porto Alegre, Brazil

**Keywords:** Mesenchymal stem cell, Senescence, Obesity, Chronic inflammation, Mitochondria

## Abstract

**Background:**

Adipose-derived stem cells (ADSC) are multipotent cells implicated in tissue homeostasis. Obesity represents a chronic inflammatory disease associated with metabolic dysfunction and age-related mechanisms, with progressive accumulation of senescent cells and compromised ADSC function. In this study, we aimed to explore mechanisms associated with the inflammatory environment present in obesity in modulating ADSC to a senescent phenotype. We evaluated phenotypic and functional alterations through 18 days of treatment. ADSC were cultivated with a conditioned medium supplemented with a pool of plasma from eutrophic individuals (PE, n = 15) or with obesity (PO, n = 14), and compared to the control.

**Results:**

Our results showed that PO-treated ADSC exhibited decreased proliferative capacity with G2/M cycle arrest and *CDKN1A* (p21^WAF1/Cip1^) up-regulation. We also observed increased senescence-associated β-galactosidase (SA-β-gal) activity, which was positively correlated with TRF1 protein expression. After 18 days, ADSC treated with PO showed augmented *CDKN2A* (p16^INK4A^) expression, which was accompanied by a cumulative nuclear enlargement. After 10 days, ADSC treated with PO showed an increase in NF-κB phosphorylation, while PE and PO showed an increase in p38MAPK activation. PE and PO treatment also induced an increase in senescence-associated secretory phenotype (SASP) cytokines IL-6 and IL-8. PO-treated cells exhibited decreased metabolic activity, reduced oxygen consumption related to basal respiration, increased mitochondrial depolarization and biomass, and mitochondrial network remodeling, with no superoxide overproduction. Finally, we observed an accumulation of lipid droplets in PO-treated ADSC, implying an adaptive cellular mechanism induced by the obesogenic stimuli.

**Conclusions:**

Taken together, our data suggest that the inflammatory environment observed in obesity induces a senescent phenotype associated with p38MAPK/NF-κB axis, which stimulates and amplifies the SASP and is associated with impaired mitochondrial homeostasis.

**Supplementary Information:**

The online version contains supplementary material available at 10.1186/s12979-023-00378-0.

## Background

Obesity is a condition that shares characteristics of age-related diseases, related to a chronic low-grade inflammatory state (inflammaging), which contributes to the development of metabolic syndrome and insulin resistance [1]. In fact, obesity and aging share multiple mechanisms related to the progression of metabolic dysregulation [2, 3], thus suggesting that obesity might accelerate the rate of aging [4, 5]. For instance, adipose tissue dysfunction is a crucial feature in the physiopathology of obesity, characterized by sustained inflammation. Moreover, the chronic and systemic pro-inflammatory state and the accumulation of senescent cells on metabolic tissues induce a harmful environment, as observed in the elderly [6–9].

Adipose-derived stem cells (ADSC) are multipotent cells present in the adipose tissue that comprise a highly proliferative niche of stem cells [10–13], a characteristic that persists in vitro. Reduced proliferative capacity is usually associated with impaired tissue regeneration and loss of stemness characteristics, factors observed during adipose tissue aging [9, 14]. In obesity, the turnover of ADSC might be accelerated, with positive and negative effects on adipose tissue. Increased turnover of ADSC can result in the expansion of adipose tissue, which can help store excess energy and prevent it from accumulating in other organs. Furthermore, the accumulation of dysfunctional adipocytes contributes to metabolic dysfunction, insulin resistance, and the appearance of senescent cells [15–18].

Cellular senescence entails a proliferative cell cycle arrest with the acquisition of a pro-inflammatory senescence‐associated secretory phenotype (SASP) [19] The presence of senescent cells has been causally associated with the appearance of multiple age-associated diseases [20]. In the context of obesity, the removal of senescent cells has proven to ameliorate insulin sensitivity, reduce macrophage homing to adipose tissue and inflammation (metaflammation), and alleviate clinical complications of diabetes and age-related adipose tissue metabolic dysfunction, as well as progenitor cell homeostasis [21, 22]. While the inflammatory milieu of adipose tissue seemed to play a crucial role in modulating ADSC to senescence [15, 23–25], little is known regarding the factors driving this phenotype.

In this regard, we aimed to explore how the inflammatory milieu, present in obesity, modulates ADSC into a senescent phenotype. We conducted a study to explore the hypothesis that chronic in vitro exposure to plasma from individuals with obesity can effectively trigger cellular senescence in ADSC. In the present study, we observed that chronic exposure to an obesogenic environment hampered ADSC proliferation and induced mitochondrial remodeling, modulating into a senescent phenotype through the activation of the p38MAPK/NF-κB axis.

## Results

### Demographic characteristics

Clinical and demographic data are described in Table 1. Individuals were clinically classified with extreme obesity or healthy eutrophic, based on BMI (p < 0.0001) and absence of metabolic syndrome. There was no difference regarding the frequency of sex (p = 0.1761) and age (p = 0.7100) between the groups.

**Table 1:** Baseline and demographic characteristics

	Groups		p value
	Eutrophic (n = 15)	Obesity (n = 14)	
Sex (male), n/total (%)	8/15 (53.3)	4/14 (28.6)	0.1761
Age (years), median (IQR)	29.0 (27.0–37.0)	34.5 (25.0–38.7)	0.7100
BMI, median (IQR)	22.1 (20.7–23.9)	49.3 (43.5–50.6)	**< 0.0001**
Physical activity, n/total (%)	0/15 (0)	0/14 (0)	**–**
Comorbidities, n/total (%)			
Type 2 Diabetes mellitus	0/15 (0)	1/14 (7.14)	**–**
Dyslipidemia	0/15 (0)	4/14 (28.6)	**–**
Hepatic Steatosis	0/15 (0)	6/14 (42.8)	**–**
Hypertension	0/15 (0)	7/14 (50.0)	**–**
Metabolic Syndrome	0/15 (0)	0/14 (0)	**–**
Medication therapy			
ACE Inhibitors (ACEI)	0/15 (0)	3/14 (21.43)	**–**
Angiotensin II receptor blockers	0/15 (0)	1/14 (7.14)	**–**
Calcium channel blockers	0/15 (0)	1/14 (7.14)	**–**
Loop diuretics	0/15 (0)	2/14 (14.28)	**–**
Statin	0/15 (0)	1/14 (7.14)	**–**
Selective beta-blockers	0/15 (0)	3/14 (21.43)	**–**
Thiazide diuretics	0/15 (0)	2/14 (14.28)	**–**

Abbreviations: ACEI: Angiotensin-Converting Enzyme Inhibitors; BMI: Body mass index; IQR: Interquartile range

Results are shown in frequency (percentage, %) or median (IQR 25–75%)

Results in bold indicate differences between groups, assessed by unpaired t-test (95% confidence interval)

### The obesogenic environment reduces ADCS proliferation with G2/M cycle arrest

**Fig. 1:**
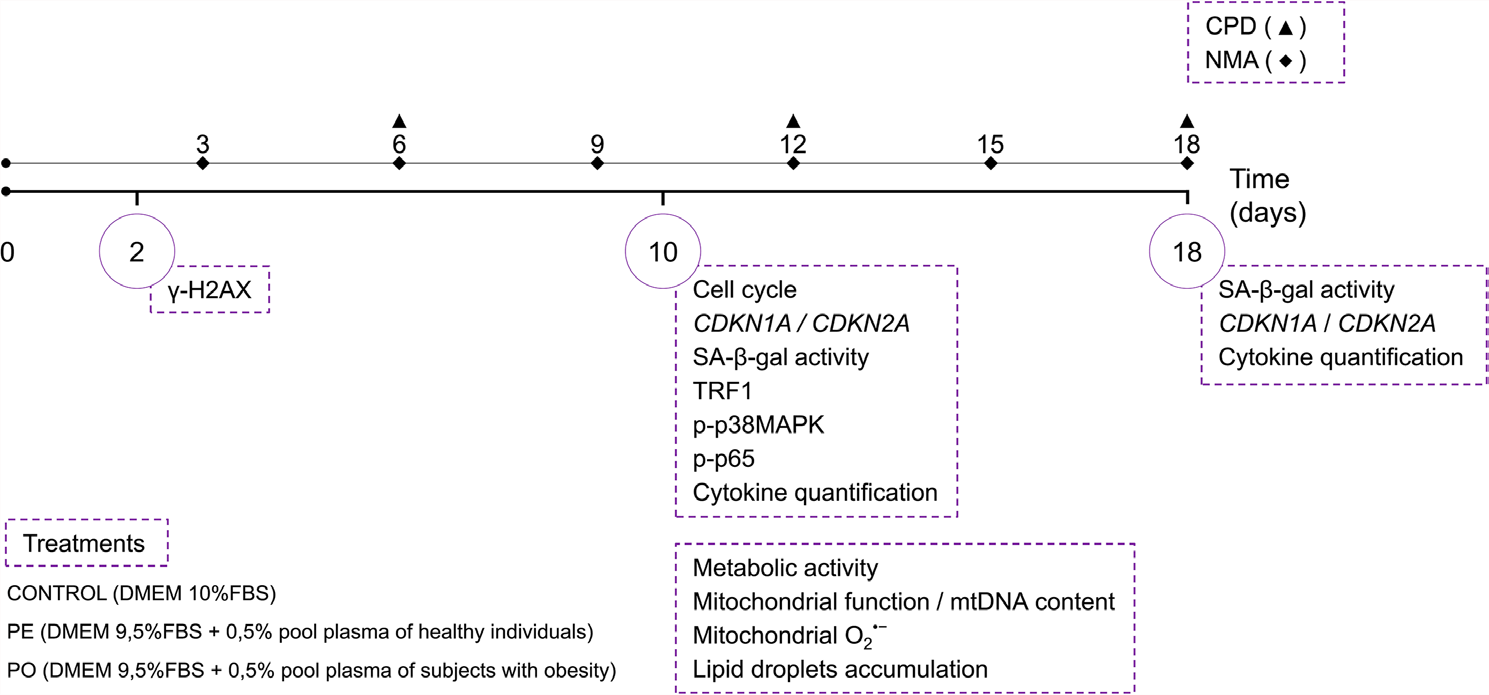
Workflow of the experimental design. Adipose-derived stem cells (ADSC) from human origin were maintained in culture media with 10% FBS (control) or treated with FBS supplemented with a pool of plasma (0.5%) from eutrophic volunteers (PE) or a pool of plasma (0.5%) from patients with obesity (PO) for 18 days. Experiments were done at different time points (solid lines 2, 10, and 18 days). CPD and NMA analysis were done every 6 (dashed line, triangle for CPD) or 3 days (dashed line, diamond for NMA). Abbreviations: SA-β-gal: Senescence-associated beta-galactosidase; *CDKN1A*: Cyclin-dependent kinase inhibitor 1 A; *CDKN2A*: Cyclin-dependent kinase inhibitor 2 A; CPD: cumulative population doubling; DMEM: Dulbecco’s modified Eagle’s medium; mtDNA: Mitochondrial DNA; NMA: nuclear morphometric analysis; O2^•−^: Superoxide anion; p-p38MAPK: phosphorylated p38 mitogen-activated protein kinase; p-p65: phosphorylated nuclear factor kappa B NF-κB p65 subunit; PE: pool of plasma from eutrophic individuals supplemented in culture medium; PO: pool of plasma from individuals with obesity supplemented in culture medium; TRF1: Telomeric repeat factor 1

To evaluate toxicity induced by plasma supplementation, we tested different concentrations obtained from both eutrophic and individuals with obesity. The results showed that the highest concentrations (1%, 1.5%, and 2%) of plasma were noxious to ADSC after culture from day 8 (p < 0.0001) (Supplementary Figure S1A). In this sense, we chose a low plasma concentration (0.5%) for the subsequent experiments. The experimental design is represented in Fig. 1.

**Fig. 2:**
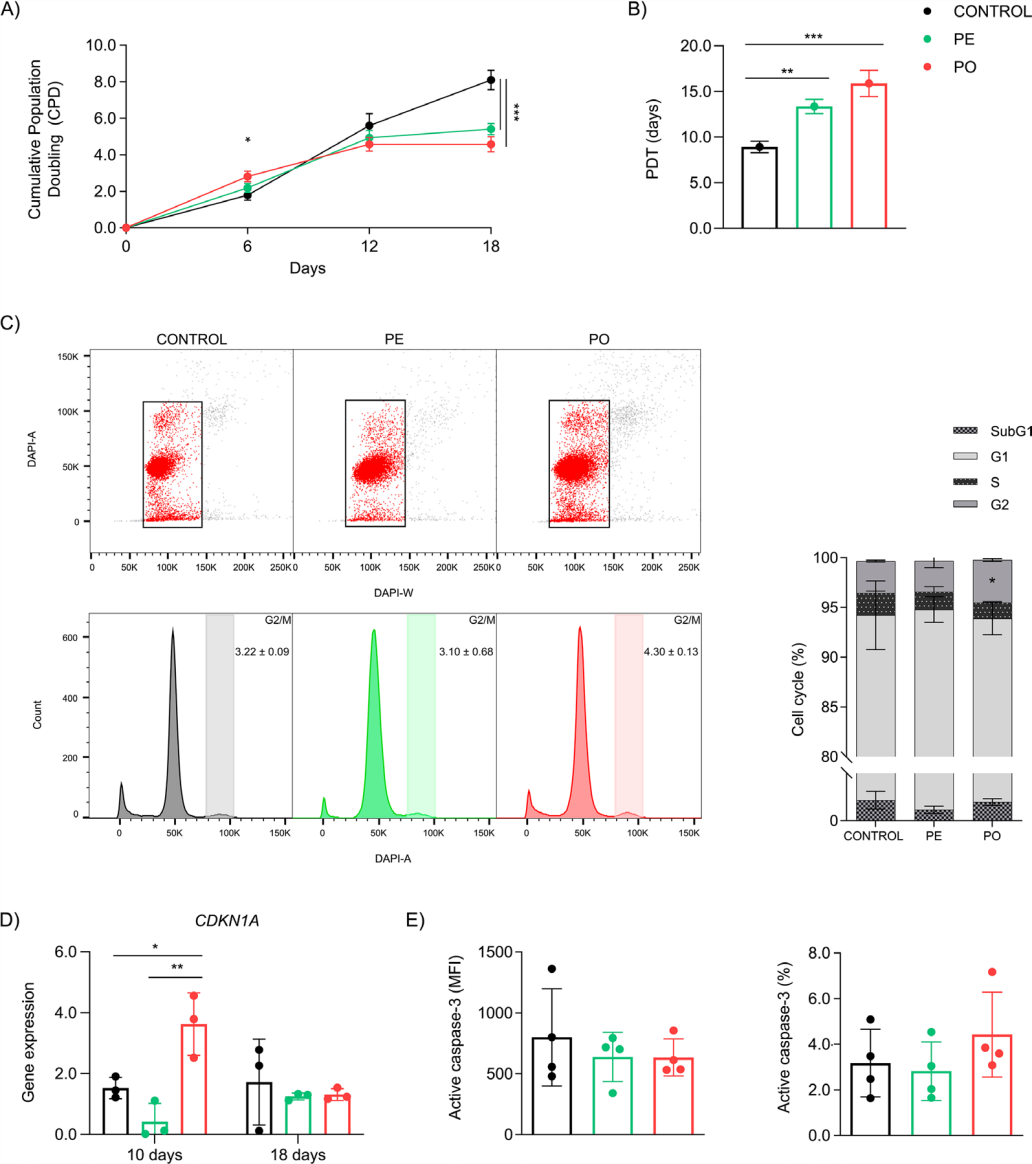
Chronic exposure to plasma from patients with obesity causes cell cycle arrest in G2/M in ADSC. (**A**) Kinetics of CPD after 18 days of plasma exposure (n = 3). (**B**) Population doubling time (PDT) showing an increase in PE and PO groups, compared to control. (**C**) (Left) Representative backgated plots and histograms from flow cytometry analysis of cell cycle and (right) quantification of cell cycle phases. The plot shows an accumulation of ADSC cells in the G2/M phase in PO treatment after 10 days. **D**) *CDKN1A* (p21^WAF1/Cip1^) mRNA expression (relative to *RPLP0* housekeeping gene) was quantified after 10 or 18 days of treatment. **E**) No difference in the (left) expression or (right) positive cells of active caspase-3 in ADSC treated with plasma or control group. Data presented as mean and standard deviation (SD). Differences were considered when p < 0.05 (*), p < 0.01 (**), p < 0.001 (***), or p < 0.0001 (****), evaluated by one-way ANOVA followed by Tukey post-test or AUC, with a confidence interval of 95%. Abbreviations: AUC: area under the curve; *CDKN1A*: Cyclin-dependent kinase inhibitor 1 A; PDT: population doubling time; PE: pool of plasma from eutrophic individuals supplemented in culture medium; PO: pool of plasma from individuals with obesity supplemented in culture medium

The kinetics of proliferation was assessed by analyzing cell density at 6, 12, and 18 days post-treatment. We observed that ADSC treated with PO had an increase in proliferation compared to control (p = 0.0086) after day 6. Interestingly, although CPD was similar between groups at day 12, after 18 days we detected that both PE and PO showed a prominent reduction in CPD (p = 0.0006 and p = 0.0001, respectively) (Fig. 2A and Supplementary Figure S1B), as well as increased population doubling time (PDT, p = 0.0042 and p = 0.0004, respectively) when compared with control (Fig. 2B). To evaluate the effect of the entire treatment on day 18, we additionally performed an integrated analysis of CPD. Our data shows a global reduction in proliferation in PE (p = 0.0114) and, to a higher extent, in PO (p = 0.0073) when compared to the control (Supplementary Figure S1C).

Because both PE and PO treatments markedly reduced ADSC proliferation, we explore the dynamics of cell cycle progression. Our data showed an accumulation of cells in the G2/M phase in PO compared to control (p = 0.0376) and PE (p = 0.0247), suggesting that the reduced proliferation of ADSC could be a consequence of a cell cycle arrest, as early observed after 10 days of treatment (Fig. 2C left and right). Cell cycle progression is regulated by cyclin-dependent kinase (CDK) inhibitors, resulting in proliferation arrest in response to cellular stress. In this sense, we evaluated the gene expression of *CDKN1A* (p21^WAF1/Cip1^) after 10 and 18 days of treatment. Our data showed *CDKN1A* up-regulation in PO compared to PE (p = 0.0037) and control cells (p = 0.0267) only at 10 days. No differences were observed at 18 days (Fig. 2D).

We then evaluate the immunocontent of active caspase-3, to assess whether this halt in population doubling was associated with the caspase-3-dependent apoptosis pathway. Our analysis revealed no differences in either expression level or percentage of cells between groups (Fig. 2E left and right). These data suggested that chronic exposure to a pro-inflammatory environment observed in obesity reduces the proliferation of ADSC by interfering with cell cycle progression and independently of caspase-3/apoptosis.

### ADSC display a progressive nuclear enlargement through obesogenic exposure

**Fig. 3:**
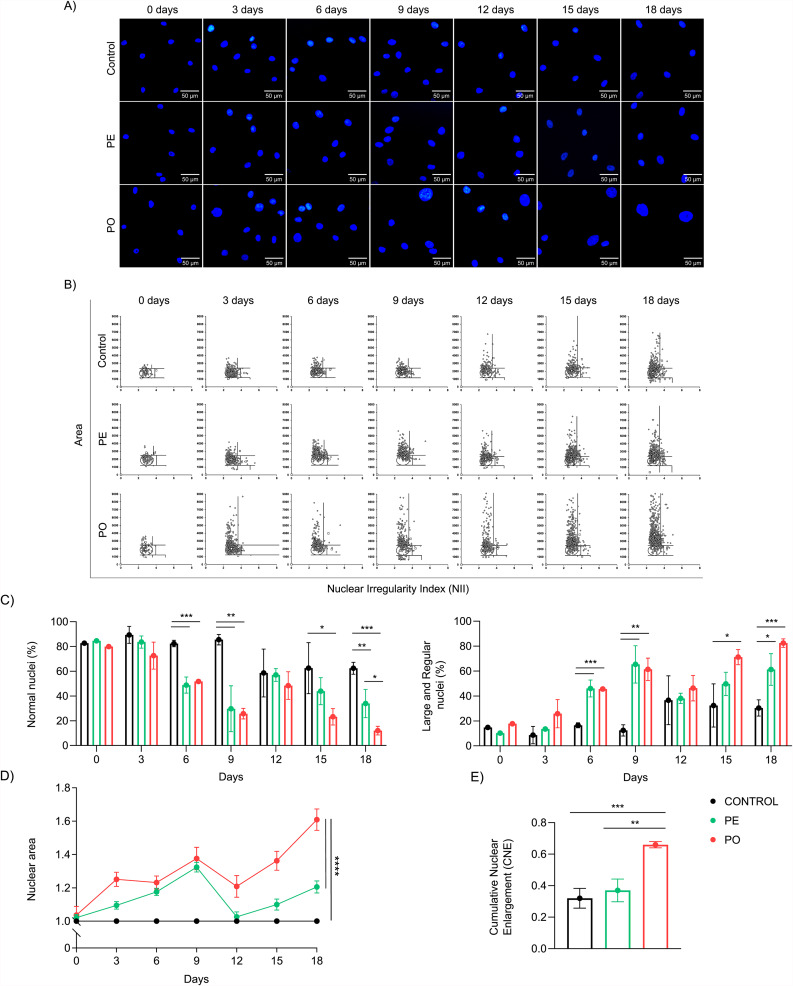
Nuclear enlargement in ADSC through obesogenic environment exposure. (**A**) Representative images (scale bar 50 μm) of ADSC cultured in control media or supplemented with PE or PO. Nuclei were stained with DAPI and images were acquired using an Olympus IX71 microscope on specified days. (**B**) (Right) Distribution of nuclei area versus NII. (**C**) (Left) Quantification (%) of normal (N) nuclei or (right) large and regular (LR) nuclei. (**D**) Relative measurement of nuclear area compared to control among treatments expressed as a fold-change. (**E**) Integrated analysis demonstrating CNE augment of ADSC nuclei in cells treated with PO compared to PE and control. Data presented as mean and standard deviation (SD). Differences were considered when p < 0.05 (*), p < 0.01 (**), or p < 0.001 (***), evaluated by one-way ANOVA test followed by Tukey post-test or area under the curve, with a confidence interval of 95%. Abbreviations: CNE: cumulative nuclear enlargement; NII: nuclear irregularity index; PE: pool of plasma from eutrophic individuals supplemented in culture medium; PO: pool of plasma from individuals with obesity supplemented in culture medium

Nuclear morphology and architecture might be affected during aging [26, 27], with reports demonstrating that increasing nuclear area can also be recognized as a cellular senescence marker [28–32]. To evaluate whether chronic exposure to plasma was accompanied by nuclear enlargement, we performed a nuclear morphometric analysis (NMA). We observed a decreased percentage of normal nuclei at 6, 9, and 18 days of treatment in both PE (p = 0.0001, p = 0.0022, and p = 0.0079, respectively) and PO (p = 0.0002, p = 0.0015, and p = 0.0004, respectively) (Fig. 3A-B C left). These alterations were accompanied by an increase in the percentage of large and regular nuclei in PE (p = 0.0003, p = 0.0019, and p = 0.0101, respectively) and PO (p = 0.0003, p = 0.0029, and p = 0.0007, respectively) (Fig. 3A-B C right). Additionally, we analyzed the magnitude of nuclear enlargement along the treatments. Our results showed that the nuclear area increased in PO throughout the 18-day treatment, compared to PE (p < 0.0001) and control cells (p < 0.0001) (Fig. 3D). Finally, when we evaluated an integrated analysis of the entire treatment, we observed an increase of cumulative nuclear enlargement (CNE) in PO compared to PE (p = 0.0018) and control (p = 0.0008) (Fig. 3E), suggesting that the obesogenic environment accelerated and potentiated nuclear enlargement, a distinctive feature of senescence.

### Plasma from patients with obesity induces senescence in ADSC and is correlated with increased TRF1 levels

**Fig. 4:**
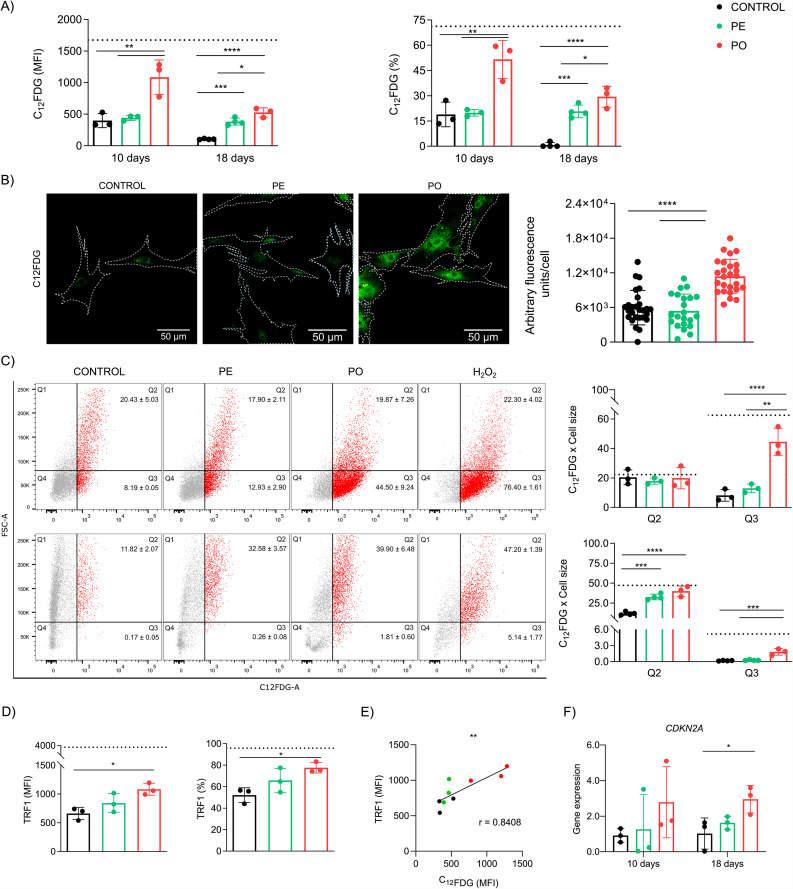
Obesogenic stimuli induce senescence and increase TRF1 levels in ADSC. (**A**) Quantitative up-regulation of SA-β-gal activity accessed by flow cytometry after 10 or 18 days showed (left) MFI and (right) percentage of cells in the PO group compared to control and PE. (**B**) (Left: scale bar 50 μm) Representative images of SA-β-gal activity measured by fluorescence microscopy in cultured cells after 10 days of treatment. Nuclei were counter-stained with Hoechst 33342. White dashed lines were manually drawn and represented the contour of cellular membranes. (Right) Fluorescence was quantified as arbitrary units/cell (n = 20–25 cells/treatment in triplicate). (**C**) (Left) Representative plots from flow cytometry analyzing SA-β-gal activity and cell size (estimated by forward scatter – FSC). (Right) Quantitative analyses (%) of Q2 and Q3 populations in 10 and 18 days of treatment. (**D**) (Left) TRF1 protein expression (MFI) and (right) percentage (%) of cells were augmented in ADSC treated with PO after 10 days. Dashed lines represent ADSC incubated with 300 μM of hydrogen peroxide (H_2_O_2_) for 3 h two days before experiments, treated as a positive control of senescent cells. (**E**) TRF1 expression and SA-β-gal activity were positively correlated after 10 days of treatment (r = 0.8408, p < 0.0001). (**F**) *CDKN2A* (p16^INK4A^) mRNA expression (relative to *RPLP0* housekeeping gene) was quantified after 10 or 18 days of treatment. Data are presented as mean and standard deviation (SD). Differences were considered when p < 0.05 (*), p < 0.01 (**), p < 0.001 (***), or p < 0.0001 (****), evaluated by one-way ANOVA test followed by Tukey post-test, with a confidence interval of 95%. Abbreviations: *CDKN2A*: Cyclin-dependent kinase inhibitor 2 A; FCS: forward scatter; PE: pool of plasma from eutrophic individuals supplemented in culture medium; PO: pool of plasma from individuals with obesity supplemented in culture medium; SA-β-gal: Senescence-associated beta-galactosidase; TRF1: Telomeric repeat factor 1

To extend our findings, we then investigated classic cellular and molecular senescence markers. Firstly, we evaluated SA-β-gal activity, a surrogate marker of senescence [33]. We observed that treatment with PO augmented SA-β-gal activity compared to control (p = 0.0066) and PE groups (p = 0.0090), as well as an increase in C_12_FDG-positive cells compared to control (p = 0.0054) and PE groups (p = 0.0063) after 10 days of culture (Fig. 4A, left and right). Similar results were observed in an independent analysis performed by confocal microscopy, which denoted increased SA-β-gal activity in PO treatment, compared to control (p < 0.0001) and PE (p < 0.0001) groups (Fig. 4B, left and right). To evaluate whether this response remained after 18 days, we observed that PO showed an increase in both SA-β-gal activity as well as C_12_FDG-positive cells in comparison with PE (p = 0.0123 and p = 0.0489, respectively) and control (p < 0.0001 and p < 0.0001, respectively). Curiously, after 18 days, PE showed increased enzyme activity (p = 0.0001) and percentage of positive cells (p = 0.0003) compared to the control group (Fig. 4A, left and right).

Since senescence is often accompanied by cell size enlargement [34, 35], we investigated whether increased SA-β-gal activity was linked to morphological changes in ADSC size by analyzing forward scatter (FSC). After 10 days of treatment, we observed that increased SA-β-gal activity observed in PO was independent of cell enlargement (p = 0.0016 compared to PE, and p = 0.0008, compared to control) (Fig. 4C, upper panel). Curiously, at 18 days of treatment, we detected that C_12_FDG-positive cells both in PO (p < 0.0001) and PE (p = 0.0003) also exhibited augmented cell size compared to the control (Fig. 4C, lower panel). These findings suggest the increased SA-β-gal activity occurs at early stages in our model, and that cell enlargement is a later acquired feature in senescent ADSC.

We have previously shown that TRF1 is a major driver of cellular senescence in PBMC [36]. To further explore the relevance in the context of obesity, we evaluated TRF1 expression in ADSC. We found that only PO treatment induced an increase in TRF1 protein expression (p = 0.0163) (Fig. 4D, left) and percentage of TRF1-positive cells (p = 0.0202) (Fig. 4D, right) compared to the control group after 10 days. PE and control cells remained similar. Interestingly, we detected a positive correlation between SA-β-gal activity and TRF1 expression (r = 0.8408, p = 0.0045) (Fig. 4E).

To better evaluate whether PO-treated ADSC achieved a permanent proliferation arrest, we assessed *CDKN2A* gene expression, which codes for protein p16^INKA4^. In agreement with our previous results, *CDKN2A* expression was increased in PO compared to control (p = 0.0355) at 18 days but no difference was observed after 10 days of treatment (Fig. 4F), denoting a persistent phenotype induced by the obesogenic milieu.

### Chronic exposure to plasma triggers inflammation and is associated with p38MAPK/NF-κB axis in senescent ADSC

**Fig. 5:**
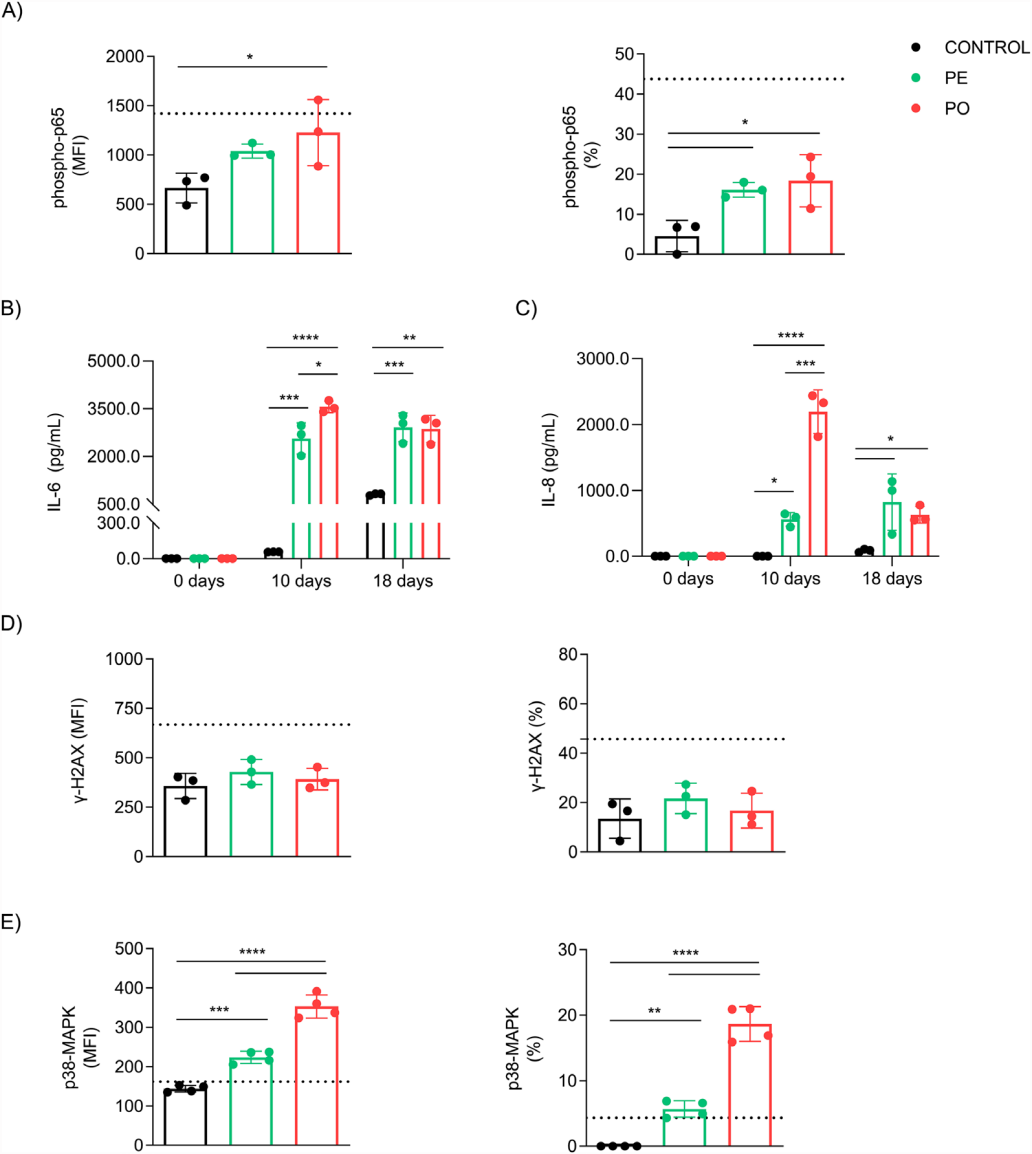
Chronic exposure to plasma triggers inflammation and is associated with p38MAPK/NF-κB axis in senescent ADSC. Flow cytometry analysis of (**A**) NF-κB p65 subunit phosphorylation levels (left, MFI, and right, %) were higher in PO when compared to control after 10 days of culture. Quantification of (**B**) IL-6 secretion and **C)** IL-8 secretion were higher in PO and PE groups in comparison to control treatment after 10 and 18 days. (**D**) γ-H2AX protein expression after 2 days (left, MFI, and right, %) of ADSC was similar among treatments with plasma or control. (**E**) p38MAPK phosphorylation levels were augmented (left, MFI, and right, %) in PO compared to both PE and control groups after 10 days of treatments. Dashed lines represent ADSC incubated with 300 μM of hydrogen peroxide (H_2_O_2_) for 3 h two days before experiments, treated as a positive control of senescent cells. Data presented as mean and standard deviation (SD). Differences were considered when p < 0.05 (*), p < 0.01 (**), p < 0.001 (***), or p < 0.0001 (****), evaluated by one-way ANOVA test followed by Tukey post-test, with a confidence interval of 95%. Abbreviations: Phospho-p38MAPK: phosphorylated p38 mitogen-activated protein kinase; Phospho-p65: phosphorylated nuclear factor NF-κB p65 subunit; PE: pool of plasma from eutrophic individuals supplemented in culture medium; PO: pool of plasma from individuals with obesity supplemented in culture medium; SASP: senescence-associated secretory phenotype

Because cellular senescence is strongly related to cytokines and chemokines secretion that comprises SASP [37, 38], we evaluated the NF-κB signaling - a major mediator of SASP [39]. We observed that PO treatment increased the levels and positive number of cells to phospho-NF-κB p65 subunit (p = 0.0440 and p = 0.0222, respectively), compared to control cells after 10 days of treatment (Fig. 5A, left and right). When we evaluated cytokine secretion at 10 and 18 days of plasma exposure. We observed that IL-6 secretion was significantly augmented in PE and PO compared to control after 10 days (p = 0.0001 and p < 0.0001, respectively) and 18 days (p = 0.0009 and p = 0.0010, respectively) (Fig. 5B). Similarly, boosted IL-8 secretion was observed in both PE and PO after 10 (p = 0.0330 and p < 0.0001, respectively) and 18 days (p = 0.0229 and p = 0.0309, respectively), compared to the control (Fig. 5C). Interestingly, at 10 days of plasma exposure, both IL-6 (p = 0.0157) and IL-8 (p = 0.0001) levels were increased in PO, compared to PE, but no difference was observed after 18 days (Fig. 5B and C). The levels of IL-1β, IL-10, TNF-α, and IL-12 secretion were below 1.0 pg/mL and considered undetectable (data not shown).

Senescence can be also induced by genotoxic stress that might trigger DNA damage response (DDR) pathways, activating checkpoints that halt proliferation [40, 41]. For this reason, we evaluated acute DDR signals by H2AX phosphorylation. Curiously, we did not observe differences in H2AX phosphorylation after 48 h in both MFI and the percentage of positive cells (Fig. 5D, left and right). This suggests that PO-induced senescence seems to be not triggered by the induction and/or recognition of DNA damage.

Finally, several studies have demonstrated that senescence and SASP can be mediated by the mitogen-activated protein kinase family member p38MAPK (MAPK14) in a DNA damage-independent manner [42, 43]. Indeed, we observed an increase in the expression and percentage of p38MAPK-positive cells in PO compared to PE (p < 0.0001 and p < 0.0001, respectively) and control cells (p < 0.0001 and p < 0.0001, respectively) after 10 days of treatment (Fig. 5E, left and right). PE also increased the activity (p = 0.0008) and p38-positive cells (p = 0.0027) compared to the control. These results suggest that p38MAPK phosphorylation is required for stress-induced senescence by the pro-inflammatory milieu observed in obesity.

### Plasma exposure impairs mitochondrial activity and morphology and is aggravated through obesogenic stimuli

**Fig. 6:**
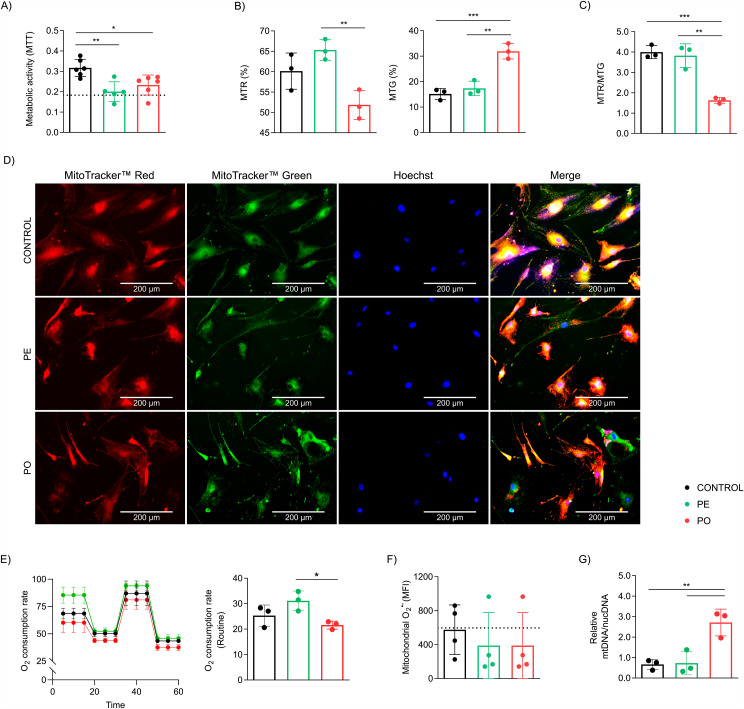
Mitochondrial metabolic activity is reduced and impairs mitochondrial function after treatment with plasma. (**A**) There was noted an energy deficiency accessed by MTT assay both in PO and PE compared to the control group after 10 days of treatment. (**B**) After 10 days of treatments, flow cytometry analysis revealed (left) decreased membrane potential (MTR), but (right) augmented mitochondrial biomass (MTG) was detected in PO compared to PE and control groups. (**C**) Decreased mitochondrial function assessed by the MTR/MTG ratio after exposure to PO when compared to PE and control treatments after 10 days. (**D**) Representative images (scale bar 200 μm) acquired by fluorescence microscopy with MTR and MTG staining after 10 days of treatment. (**E**) (Left) High-resolution respirometry was performed and (right) decreased oxygen consumption was observed in ADSC treated with PO compared to the PE group. (**F**) There was no difference in O2^•−^ expression after 10 days of treatments. (**G**) Relative mtDNA copies were increased in PO after 10 days of treatment. Data presented as mean and standard deviation (SD). Dashed lines represent positive control of senescence assessed by 300 μM of hydrogen peroxide (H_2_O_2_) treated for 3 h two days before experiments. Differences were considered when p < 0.05 (*), p < 0.01 (**), p < 0.001 (***), or p < 0.0001 (****), evaluated by one-way ANOVA test followed by Tukey post-test, with a confidence interval of 95%. Abbreviations; MFI: median fluorescence intensity; ; mtDNA: mitochondrial DNA; MTG: MitoTracker™ Green FM; MTR: MitoTracker™ Red CMXRos; MTT: [3-(4,5-Dimethylthiazol-2-yl)-2,5-diphenyltetrazolium bromide]; nucDNA: nuclear DNA; ; PE: pool plasma of eutrophic individuals supplemented in the complete culture medium; PO: pool plasma of individuals with obesity supplemented in the complete culture medium; O2^•−^: Mitochondrial superoxide

Mitochondria are complex interconnected organelles resulting in a dynamic mitochondrial network. Our results demonstrated reduced metabolic activity, assessed by the MTT assay, in both PE and PO treatments when compared to control cells (Fig. 6A, p = 0.0027 and p = 0.0185, respectively). We also observed that exposure to PO treatment induced a decrease in mitochondrial membrane potential (MTR staining) compared to PE (p = 0.0088) (Fig. 6B, left) and augmented mitochondrial biomass (MTG staining) compared to PE and control groups (p = 0.0014 and p = 0.0006, respectively) (Fig. 6B, right). Furthermore, we observed a decrease in mitochondrial function, assessed by the ratio between membrane potential and mitochondrial biomass (MTR/MTG), in ADSC treated with the PO plasma in comparison to PE and control treatments (p = 0.0012 and p = 0.0008, respectively) (Fig. 6C). Representative images of independent confocal analysis are shown in Fig. 6D.

Because cellular senescence can be related to mitochondrial and/or metabolic dysfunction [44, 45], we evaluated dynamical mitochondrial function by high-resolution respirometry. Our results demonstrated reduced O_2_ consumption in routine respiration of PO-treated cells (p = 0.0314) compared only to PE, after 10 days of treatment (Fig. 6E, left and right). The other aspects of mitochondrial respiration evaluated remained unchanged despite the treatments applied (Supplementary Figure S2A-E). Since we observed changes in mitochondrial function related to PO treatment, we investigated whether PO could induce ROS overproduction. Interestingly, we did not observe any differences in mitochondrial superoxide (O_2_^•−^) expression (Fig. 6F), suggesting that the loss of mitochondrial function is not associated with increased mitochondrial O_2_^•−^ production.

Mitochondrial DNA (mtDNA) copy number can be modified as compensation for organelle damage [46, 47]. In this sense, we evaluated the relative quantification of the mitochondrial to nuclear DNA copies (mtDNA/nucDNA). Our results demonstrated augmented relative mtDNA in PO compared to PE (p = 0.0082) and control groups (p = 0.0070) after 10 days of plasma treatment (Fig. 6G).

**Fig. 7:**
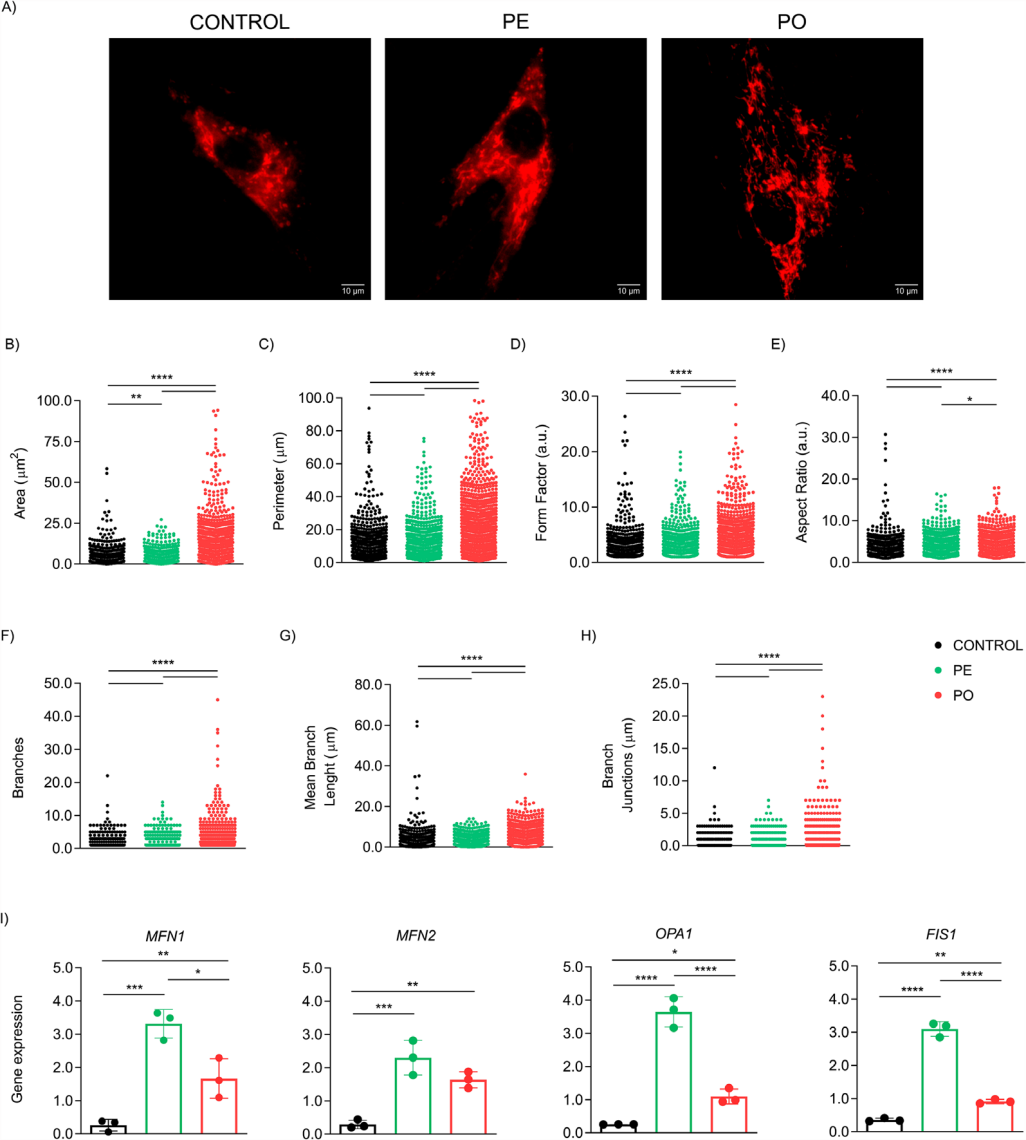
Plasma stimuli promote mitochondrial morphology and network remodeling. Cell-live imaging analysis in ADSC exposed to the plasma altered mitochondria morphology after 10 days of treatment. (**A**) Representative images analyzed with MitoTracker Red™ Red CMXRos staining (scale bar: 10 μm) using Mitochondria Analyzer plugin from ImageJ FIJI. Analysis was performed in 10–20 cells (treatment in triplicate) acquired from different fields. The following measurements are presented (**B**) Area (μm^2^); (**C**) Perimeter (μm); (**D**) Form Factor (a.u.); (**E**) Aspect Ratio (a.u.); (**F**) Branches/mitochondria; (**G**) Mean Branch Length (μm); and (**H**) Branch Junctions (μm). (**I**) Gene expression (relative to *HPRT1* housekeeping gene) of mitochondrial fusion (MFN1 and MFN2 and OPA1) and fission (FIS1) components were up-regulated both in PE and PO compared to control. Data presented as mean and standard deviation (SD). Differences were considered when p < 0.05 (*) or p < 0.0001 (****), evaluated by one-way ANOVA test followed by Tukey post-test, with a confidence interval of 95%. Abbreviations: a.u.: arbitrary units; FIS1: Mitochondrial Fission Protein 1; MFN1: Mitofusin 1; MFN2: Mitofusin 2; OPA1: Optic Atrophy 1; PE: pool plasma of eutrophic individuals supplemented in complete culture medium; PO: pool plasma of individuals with obesity supplemented in the complete culture medium;

To explore whether mitochondrial morphology could be affected by plasma exposure, we performed additional analysis in live-cell imaging after 10 days of treatment (Fig. 7A). We quantified aspects related to mitochondria complexity and network. Notably, our analysis revealed significant alterations when comparing ADSC treated with plasma (PE and PO) and control groups. While plasma treatment itself demonstrated a significant capacity to induce shifts in mitochondrial morphology compared to the control group, it is noteworthy that the obesogenic environment exhibited even more pronounced alterations. Specifically, we observed a prominent increase in both mitochondrion area (p < 0.0001) and perimeter (p < 0.0001) (Fig. 7B and C). Moreover, the shape of the mitochondria was significantly altered in the PO group, as indicated by the form factor (p < 0.0001 compared to both groups) and aspect ratio (p < 0.001 compared to control; p = 0.0425 compared to PE) (Fig. 7D and E). Additionally, our analysis demonstrated enhanced mitochondrial network connectivity in the PO group compared to both control and PE groups, as evidenced by increased branches (p < 0.0001), mean branch length (p < 0.0001), and branch junctions (p < 0.0001) (Fig. 7F-H). Our results suggest alterations in the mitochondrial network and morphology, which may indicate an impairment of mitochondrial function. These alterations appear to be aggravated in the context of obesity. We also evaluated the dynamics of the mitochondrial life cycle, by assessing the expression of genes related to mitochondrial fusion and fission. We observed that fusion proteins MFN1, MFN2 and OPA1, as well as fission protein FIS1, were up-regulated both in PE (p = 0.0003, p = 0.0009, p < 0.0001, and p < 0.0001 respectively) and PO (p = 0.0182, p = 0.0068, p = 0.0288, and p = 0.0057 respectively) compared to control (Fig. 7I).

Taken together, our results reveal alterations in the mitochondrial network and morphology and suggest an impairment of mitochondrial function that seems to be aggravated in the context of obesity.

### The obesogenic environment induces the accumulation of lipid droplets

**Fig. 8:**
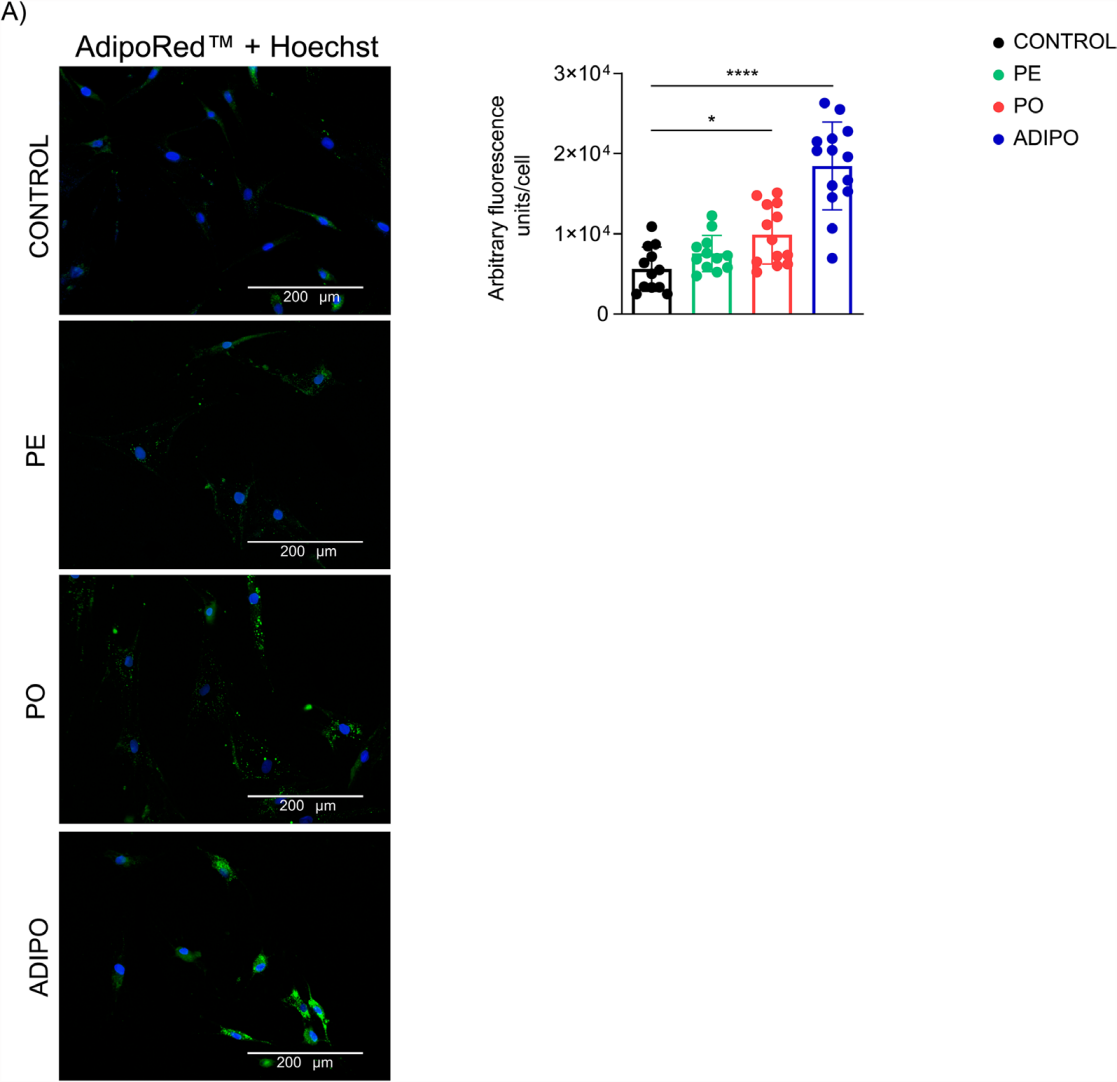
The obesogenic environment induces the accumulation of lipid droplets. (Left: scale bar 200 μm) Image analysis showed that there was an increase in the accumulation of lipid droplets of ADSC exposed to the obesogenic environment for 10 days compared to the control group. We included a group composed of ADSC induced to adipogenic differentiation as a positive control (ADIPO). (Right) Fluorescence was quantified as arbitrary units/cell (n = 10–15 cells/treatment in triplicate). Data presented as mean and standard deviation (SD). Differences were considered when p < 0.05 (*) or p < 0.0001 (****), evaluated by one-way ANOVA test followed by Tukey post-test, with a confidence interval of 95%. Abbreviations: PE: pool plasma of eutrophic individuals supplemented in the complete culture medium; PO: pool plasma of individuals with obesity supplemented in the complete culture medium; ADIPO: ADSC induced to adipogenic differentiation for 10 days

Since plasma is composed of important factors for the differentiation of adipocyte precursor cells (such as growth hormone, insulin, and other molecules that promote adipogenic differentiation), we also evaluated the ability of ADSC to differentiate into preadipocytes by lipid droplet accumulation. After 10 days of treatment, we observed increased content of lipid droplets in the PO group compared to the control group (p = 0.0365), but not in the PE (Fig. 8A). These results suggest that the mechanism of cellular adaptation observed by plasma treatment is in part related to the obesogenic environment.

## Discussion

Obesity-associated inflammatory mechanisms play a key role in the pathogenesis of metabolic-related diseases [48], and adipose tissue plays a role in the development of a systemic inflammatory state that contributes to the appearance of obesity-associated cardiovascular disease [49]. In this regard, ADSC are a useful tool for studying tissue regeneration and immunomodulation therapies due to their cellular plasticity and ease of manipulation. Because they can change their identity or function in response to different stimuli, ADSC can be used to elucidate mechanisms involved in various disorders, such as chronic pro-inflammatory diseases and tissue engineering in translational medicine [50, 51].

Imbalanced adipose tissue expansion and increased pro-inflammatory secretion are implicated in the premature senescence of ADSC [14, 18, 25] suggesting that the accumulation of senescent cells is a root for adipose tissue dysfunction and inflammation, which may be compromised early in childhood obesity onset [52]. In the present study, we demonstrate that chronic exposure to the obesogenic pro-inflammatory environment induced the appearance of adipose-derived senescent stem cells, mimicking tissue dysfunction as a key factor in the pathophysiology of obesity-related chronic metabolic diseases.

A major feature of cellular senescence is cell cycle arrest [53, 54], mainly through activation of the p53/p21^WAF1/Cip1^ [55–57] and p16^INK4A^/Rb [58–60] metabolic pathways. Our results revealed an early accumulation of cells in G2/M in the PO group that was accompanied by increased expression of *CDKN1A* (p21^WAF1/Cip1^). These findings are in agreement with studies that suggest cycle arrest mediated by p21^WAF1/Cip1^ through G2/M arrest [61, 62] is important to the onset of senescence [56, 61, 63, 64]. On the other hand, studies have shown that ADSC from patients with obesity exhibits increased expression of p16^INK4A^ and SASP components IL-6 and MCP-1 that potentially promote senescent cell accumulation in vitro [17]. Interestingly, although we did not observe any difference at 10 days, after 18 days of treatment we observed an up-regulation of *CDKN2A* (p16^INK4A^) in conjunction with an increased population doubling time, suggesting a long-term arrest, and senescence perpetuation [65]. In line with our findings, a recent study highlighted the accumulation of p21-positive cells from visceral adipose tissue earlier than p16-positive cells in the context of obesity. Intriguingly, p21-expressing cells alone were found to be sufficient to induce insulin resistance. Moreover, targeting the NF-κB pathway demonstrated significant efficacy in mitigating the metabolic dysfunction induced by obesity in mice [64]. On the other hand, the similar behavior in the PE group suggests that plasma *per se* is sufficient to promote cycle arrest by additional pathways.

Another hallmark of the senescent phenotype is the increase of SA-β-gal activity in non-phagocytic cells, which is observed in obesity and aged in vitro ADSC [53, 66, 67]. Our results revealed that treatment with plasma from patients with obesity promoted a prominent increase of SA-β-gal activity after 10 and 18 days of treatment, denoting an accelerated and persistent modulation of senescence. Curiously, the plasma of eutrophic subjects also showed an increase in SA-β-gal activity at day 18, although lower than the PO group, suggesting that some components of plasma might induce an intermediate state senescence-like response. In addition, we also detected an increase in TRF1 protein expression in the PO group after 10 days of treatment and found a positive correlation between TRF1 and SA-β-gal activity. Recently, we demonstrated a negative association between the expression of this shelterin component and telomere length in PBMC within the context of obesity. Additionally, we identified TRF1 as a potential marker of premature aging [36]. Together, these findings suggest that the sustained senescent phenotype observed at 18 days, might be initiated through cell cycle arrest early at 10 days and was accompanied by a distinct modulatory phenotype associated with premature senescence in ADSC.

Morphological alterations in nuclear architecture and morphology represent central and conserved elements in the progression of cellular aging [26]. Loss of nuclear envelope protein Lamin B1, chromatin remodeling due to the accumulation of DNA damage foci, and genomic instability are mechanisms involved in nuclear size enlargement, which might be connected to stress pathways stimulating the SASP [68–71]. When evaluating the kinetics of morphometric changes in the nuclei, we observed a prominent increase in cumulative nuclear enlargement in the PO group, consistent with the progression of the senescent phenotype evaluated by the aforementioned markers. While plasma *per se* promotes an increase in the proportion of senescent cells, denoted by the kinetics of the PE group, the reduced cumulative rate suggests a mild effect compared to the obesogenic environment.

Adequate mitochondrial function is required to sustain energy demand during ADSC proliferation and regeneration [72]. Thus, an accumulation of damaged mitochondria associated with chronic inflammation could contribute to the senescent phenotype, as seen observed in obesity [73–77]. In fact, our results depicted a deficiency in basal mitochondrial energy metabolism accompanied by an increase in both mitochondrial biomass and depolarization. While the gene expression related to both fusion and fission was elevated in PE, which suggests an intense network remodeling, it is noteworthy that ADSC treated with PO displayed elongated, hyperfused, and branched morphology. These findings are in line with previous reports showing that senescent cells also exhibit a distinctive mitochondrial network with elongated and branched mitochondria [78, 79]. Moreover, increased mitochondrial mass and biogenesis are associated with elongated and hyperfused mitochondria which may modulate senescence [80, 81]. Furthermore, although we observed a metabolic shift in mitochondrial function, the senescence state appears to be independent of ROS overproduction. Similar to others, our data suggest that senescence might occur in ROS-independent mitochondrial damage [44, 45]. These data suggest that a noxious environment induced by plasma from individuals with obesity could modulate mitochondrial remodeling. Our results are also in line with Pérez et al., relating impaired function and mitochondrial content of ADSC in the context of obesity, with physiological changes in energetic metabolism [82].

The stress-activated p38MAPK pathway mediates important intracellular mechanisms related to the pathophysiology of age-related diseases, such as inflammaging and the SASP through up-regulation of both TP53 and p16^INK4A^/Rb arrest pathways [83–85]. In the present work, we detected an increase in the activation of p38MAPK in both plasma treatments after 10 days, with this effect notably more pronounced in cells exposed to the obesogenic environment. We speculate that these stimuli may contribute to the chronic activation of p38MAPK promoting senescence in ADSC. Our assumption is partially supported by studies demonstrating p38MAPK-associated senescence by chronic exposure to SASP [42, 43, 86]. A recent study showed the multifaceted functions of p38MAPK in the aging process in hematopoietic stem cells, including immune response and stemness modulation [87]. Additionally, our findings are supported by recent studies in mice, which demonstrate that obesity triggers significant changes in the ADSC secretome and proteome, leading to adipose tissue dysfunction, and potentially promoting senescence in ADSC from visceral white adipose tissue [88, 89]. Furthermore, the chronic inflammation observed in obesity may be sustained through the persistent activation of p38MAPK and NF-κB signaling and the secretion of the pro-inflammatory cytokines IL-6 and TNFα [90]. Taken together, our data suggest that the senescence phenotype observed early at 10 days was accompanied by SASP up-regulation with activation of the p38MAPK/NF-κB axis.

The presence of senescent ADSC in visceral adipose tissue secreting pro-inflammatory and pro-aging factors might drive obesity-related metabolic dysfunction. We detected that ADSC exposed to PO stimulated increased lipid droplet accumulation. These data are in line with findings demonstrating that dysregulated lipid metabolism homeostasis is associated with cells with high SA-β-gal activity, suggesting that lipid droplet accumulation might contribute to the senescent phenotype [91, 92] and a mechanism of cellular adaptation consequent to the stress-induced pro-inflammatory response. In this regard, impaired metabolic function, increased senescent cell burden, and infiltration of immune cells followed by polarization toward a pro-inflammatory phenotype, ultimately lead to a chronic and systemic low-grade inflammatory state, termed metaflammation, compared to the acute inflammatory response [93]. Metaflammation and cellular senescence can spread inflammation and premature senescence to multiple organs, contributing to systemic physiological decline [1, 93]. Chronic inflammation is associated with aging and plays a causative role in several age-related diseases such as cancer, atherosclerosis, and metabolic disorders [6, 93]. To our knowledge, this is the first study to elucidate mechanisms associated with the kinetics of cellular senescence in ADSC exposed to the complex composition of plasma from obesity.

Our group recently demonstrated the deleterious effects of this toxic peripheral environment on the immunological compartment, regarding its pro-oxidant status, efficient to induce biomolecular damage with an adaptive antioxidant response insufficient to prevent accelerated telomere shortening [36, 94]. We have also previously shown cumulative immunosenescence markers associated with mitochondrial dysfunction and reduced ATP-linked oxygen consumption rate on PBMC [95]. Therefore, these results not only corroborate the supposed obesity accelerated aging but also highlight that the pro-inflammatory milieu is an important senescence driver in ADSC in the context of obesity [93].

Although the results obtained in this work are promising and contribute to the elucidation of the mechanisms that relate to the senescent phenotype in the context of obesity, our study has limitations. The presence of multimorbidity is characteristic of obesity, and due to its heterogeneity among donors, can contribute to the emergence of confounding effects. Secondly, given the limited sample size used in this work, confounding factors may have contributed in a different way to the modulation of the senescent phenotype. Finally, we evaluated the deleterious effects of plasma as a complex setting and characteristic of obesity, so we did not explore data regarding single components.

## Conclusions

**Fig. 9:**
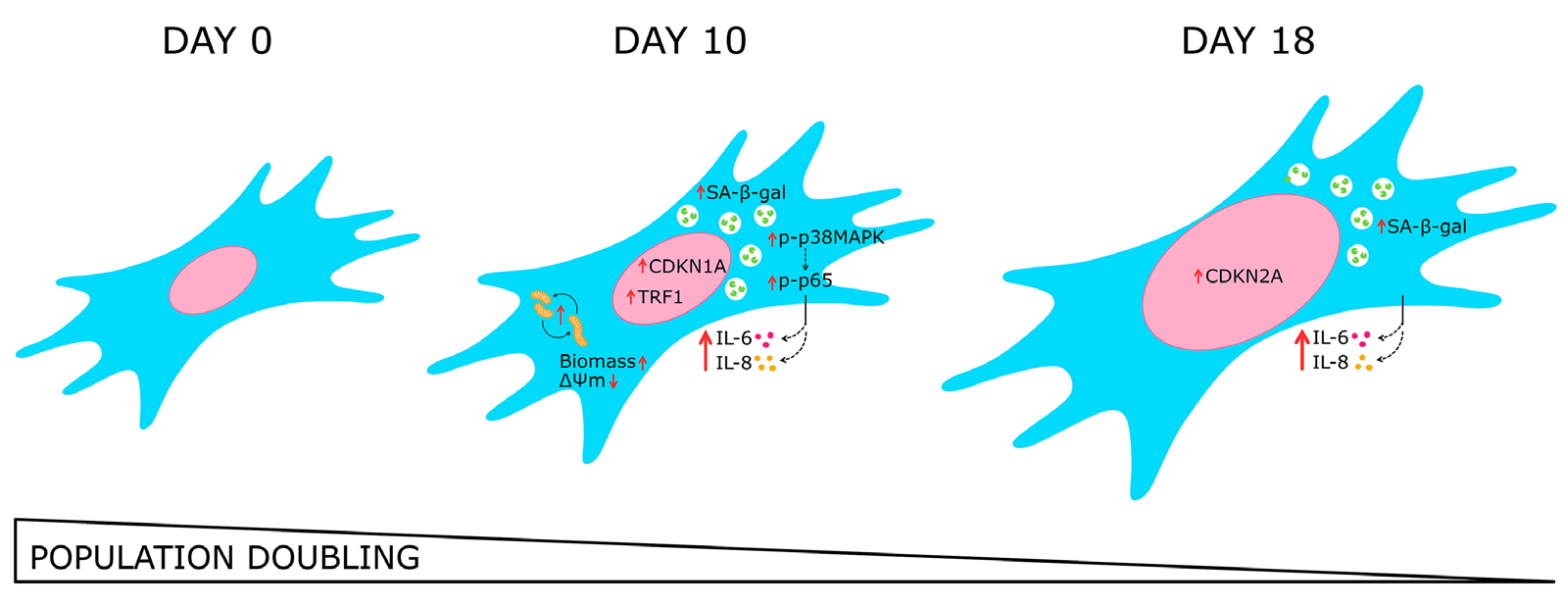
Chronic exposure to obesogenic milieu modulates adipose-derived stem cells (ADSC) to a senescent phenotype associated with the p38MAPK/NF-kB axis. In our model, the inflammatory environment observed in obesity triggers a senescent phenotype in ADSC. The exposure to obesogenic milieu stimulated an increase in SASP cytokines IL-6 and IL-8 through p38MAPK/NF-kB activation, as well as promoted mitochondrial remodeling. ADSC exhibited G2/M cycle arrest and up-regulation of *CDKN1A* (p21^WAF1/Cip1^), along with increased SA-β-gal activity positively correlated with TRF1 protein expression at day 10. After 18 days, ADSC showed persistent cycle arrest by increased *CDKN2A* (p16^INK4A^) expression, cumulative nuclear enlargement, and SASP amplification. These findings highlight the phenotypic and functional alterations in ADSC exposed to plasma from obesity and suggest a link between obesity-induced inflammation and cellular senescence

Taken together, our results highlight that the pro-inflammatory environment induced in the obesity context is associated with the activation of cellular and molecular responses that triggered, in early stages, the senescent phenotype (Fig. 9), similar to what is observed in obesity and physiological aging. Our results are in agreement with previous work, emphasizing the impact of cellular senescence on the contribution of inflammation related to obesity [6, 14, 17, 36, 42, 96]. Senescence of adipose tissue must be seen as a therapeutic target for treating aging- and obesity-related metabolic disorders. The targeting of senescent cells might be a therapeutic approach for reducing pro-inflammatory responses.

## Methods

### Subjects and biological samples

Individuals aged between 18 and 65 years were included and classified based on body mass index (BMI) and the presence of comorbidities associated with extreme obesity (BMI ≥ 40.0 kg/m²) without metabolic syndrome. Volunteers were recruited in the intraoperative period of bariatric surgery at the Centro de Obesidade e Síndrome Metabólica (COM) of Hospital São Lucas, Pontifícia Universidade Católica do Rio Grande do Sul (HSL-PUCRS). We also recruited 15 healthy eutrophic subjects (BMI = 18.5 kg/m² − 24.9 kg/m²) who underwent routine exams and agreed to participate (Table 1).

A total of 10 mL of peripheral blood was drawn by venipuncture. The blood was placed in tubes containing 5% EDTA and centrifuged at 400 xg for 15 min at room temperature. The upper fraction, corresponding to plasma, was collected, and stored at -80 °C.

### Cell culture

The human ADSC were acquired at first passage from LONZA (PT-5006, USA). The cells were cultured as specified by the manufacturer. Basically, cells were maintained in Dulbecco’s modified Eagle culture medium (DMEM) complete with low glucose concentration (1.0 g/L) supplemented with 10% fetal bovine serum (FBS), 1% penicillin/streptomycin, and 0.25 g of amphotericin B, and maintained at 37 °C with 5% CO_2_ in a humidified incubator.

For the experiments, the plasma from participants was mixed to obtain a pool of plasmas from eutrophic individuals (n = 15) and a pool of plasmas from individuals with obesity (n = 14). ADSC were cultured and divided into 3 groups: Control group (DMEM 10% FBS), PE group (DMEM 9.5% FBS supplemented with 0.5% of the plasma pool of eutrophic individuals), and PO group (DMEM 9.5% FBS supplemented with 0.5% of the plasma pool of individuals with extreme obesity). The plasma samples used in this study were matched by sex and age (Table 1). The experiments were performed when cell cultures were in the exponential growth phase, between passages 6 and 7. During cultures, 50% of the culture medium was changed every 3 days. At the end of the treatments, cells were collected by trypsinization (0.05% Trypsin 1:250 in 0.02% EDTA) for assays (Fig. 1).

### Cell proliferation by imaging cytometry

StainFree™ cell detection technology was used to evaluate toxicity for plasma supplementation by cell confluence analysis. The imaging cytometry assay was performed using the SpectraMax i3 plate reader coupled to the MiniMax 300 imaging cytometer (Molecular Devices, USA) with the optical imaging module and transmitted light configuration. Briefly, 4 × 10^2^ ADSC were seeded in 96-well plates, and treatment was started after 24 h. Confluence and proliferation data were recorded. Results were expressed as a percentage (%) of confluence along 18 consecutive days of treatments.

### Cumulative population doubling

Cell proliferation was assessed kinetically by cumulative population doubling (CPD) along 18 days of treatments and evaluated by image analysis using 300 nM DAPI (Thermo Fisher Scientific, USA) staining. The images were acquired using an Olympus IX71 fluorescent microscope (Olympus Corporation, Japan) and nuclei count was performed using Image Pro Plus 6.0 software (Media Cybernetics, USA). Cell number and CPD were calculated through the formula CPD=(LogNf–LogNi)/Log(2), where Nf was the final number of cells and Ni was the initial number of cells in a given interval of time. Population doubling time (PDT) was calculated using the formula PDT = Ln(2)/growth rate, where the growth rate was determined by Ln(Nf/Ni)/time. The CPD was then plotted versus the time (days) of culture.

### Cell cycle

At the end of the tenth day of treatment, 1 × 10^5^ ADSC were fixed with 2% paraformaldehyde for 15 min and subsequently permeabilized with 0.25% Triton X-100 supplemented with 1% FBS for 15 min. Staining with 300 nM DAPI for 10 min at room temperature was done protected from light. Then, cells were washed once with PBS 1X, and 20,000 events were acquired at a low flow rate using FACSCanto™ II (BD Biosciences, USA). The data was analyzed using FlowJo™ v10.8 software (BD Life Sciences, USA) and expressed as a percentage (%) of the population in each phase of the cell cycle.

### Senescence-associated β-galactosidase assay

Senescence was evaluated by SA-β-gal activity with the fluorogenic substrate C_12_FDG (5-dodecanoylaminofluorescein di-β-D-galactopyranoside), as previously described [97]. Briefly, 1 × 10^5^ cells were washed once with PBS 1X and incubated with 100 μM chloroquine for 1 h to induce lysosomal alkalinization, and subsequently incubated with 33 μM C_12_FDG for 2 h in a humidified incubator at 37 °C, 5% CO_2_. As a positive control, cells were treated with 300 μM hydrogen peroxide (H_2_O_2_) for 3 h two days before the experiment [98]. Cells were collected and washed with PBS 1X, and 10,000 events were acquired using FACSCanto™ II (BD Biosciences, USA). The data was analyzed using FlowJo™ v10.8 software (BD Life Sciences, USA) and expressed as median fluorescence intensity (MFI) or percentage (%).

### Nuclear morphometric analysis (NMA)

The nuclear morphometric analysis was evaluated kinetically along 18 days of treatments with 300 nM DAPI (Thermo Fisher Scientific, EUA) staining. Data analysis was performed by immunofluorescence in images acquired using an Olympus IX71 microscope (Olympus Corporation, Japan), and nuclear parameters were analyzed using Image Pro Plus 6.0 software (Media Cybernetics, USA). Cell viability and cell viability-related parameters (aspect, area/box, radius ratio, and roundness) were combined to generate the Nuclear Irregularity Index (NII) and classified based on quadrant division [30].

### Detection of intracellular proteins by flow cytometry

Intracellular proteins were detected in ADSC by staining with anti-TRF1 conjugated with Alexa Fluor-647 (clone G-7, 1:30, Santa Cruz Biotechnology, USA) and anti-active caspase-3 conjugated to PE (clone C92-605, 1:20, BD Biosciences, USA). Phosphorylated proteins were determined using anti-phospho-p38MAPK conjugated to Alexa Fluor-647 (clone pT180/pY182, 1:10, BD Biosciences, USA), anti-phospho-H2AX conjugated to Alexa Fluor-488 (pS139, clone N1-431, 1:20, BD Biosciences, USA), and anti-phospho-p65 conjugated to BV421 (pS529, clone K10-895.12.50, 1:50, BD Biosciences, USA).

The staining protocol consisted of cell fixation by CytoFix Fixation Buffer (BD Biosciences, USA) for 20 min at 4 °C. Cells were then permeabilized for 30 min with Perm Buffer III for TRF1, H2AX, and p38MAPK or with Perm/Wash 1X for p65 and active caspase-3 and washed twice in staining buffer. Next, cells were stained with corresponding fluorochrome-labeled antibodies in a staining buffer and incubated for 30 min at 4 °C protected from light. Finally, cells were washed with PBS 1X, and 10,000 events were acquired using FACSCanto™ II (BD Biosciences, USA). Data were analyzed using FlowJo™ v10.8 software (BD Life Sciences, USA) and expressed as MFI or percentage (%).

Flow cytometry data are presented according to the Minimum Information about a Flow Cytometry Experiment (MIFlowCyt) standard, as recommended by the International Society for Advancement of Cytometry (ISAC) [99].

### Metabolic activity assay

Metabolic activity was determined by [3-(4,5-Dimethylthiazol-2-yl)-2,5-diphenyltetrazolium bromide] (MTT) reduction by NAD(P)H-dependent cellular oxidoreductase enzymes (Sigma Aldrich, USA). Briefly, after 10 days of treatment (or 300 μM H_2_O_2_ for 3 h as positive control), ADSC were incubated with MTT solution (0.2 mg/ml) for 2 h at 37 °C/5% CO_2_ humidified incubator. After removing the medium, DMSO was added to dissolve formazan crystals, and the absorbance was measured at 570 and 630 nm spectrophotometrically using a microplate reader (Anthos Zenyth 340, Biochrom, UK).

### Determination of reactive oxygen species levels

To assess mitochondrial superoxide anion (O2^•−^) production, live cells were stained with the MitoSOX™ Red probe (Molecular Probes, USA). At the end of a 10-day treatment, cells were trypsinized, washed in PBS 1X and labeled with 5 μM MitoSOX™ Red probe and incubated at 37 °C for 10 min, washed again with PBS 1X, and analyzed. Data were acquired using FACSCanto™ II (BD Biosciences, USA), and analysis was performed by MFI and percentage (%) of the population, using FlowJo™ software v10.8 (BD Life Sciences, USA).

### Mitochondrial function analysis

The mitochondrial membrane potential and biomass were evaluated using MitoTracker™ Red CMXRos (MTR) (Thermo Fisher, USA) and MitoTracker™ Green FM (MTG) dye (Thermo Fisher, USA), respectively. After 10 days of treatment, 1 × 10^5^ ADSC were removed from the culture plate by trypsinization, washed with PBS 1X, and incubated at 37 °C/5% CO_2_ for 30 min with staining solution containing 100 nM MTR and 100 nM MTG in DMEM FBS-free media. Cells were then washed with PBS 1X, and 10,000 events were acquired using FACSCanto™ II (BD Biosciences, USA). The data were analyzed using FlowJo™ software v10.8 (BD Life Sciences, USA) and expressed as MFI or percentage (%) of the population. High-resolution respirometry was performed using the Oroboros O2k oxygraph (Oroboros Instruments®, Innsbruck, Austria). The protocol consisted of basal respiration assessment followed by 2 μL of 1 mg/mL oligomycin (Sigma Aldrich, Brazil) injection to evaluate the proton leak. Next, 1 mM carbonyl cyanide-4-(trifluoromethoxy) phenylhydrazone (FCCP) (Sigma Aldrich, Brazil) was titrated in 1 μL steps to observe maximum respiration. Finally, 1 μL of 1 mM rotenone (Sigma Aldrich, Brazil) and 1 μL of 5 mM antimycin A (Sigma Aldrich, Brazil) were applied together at the end of the run to observe extra-mitochondrial O_2_ consumption [100]. The chamber temperature was set at 37 °C, and 750 rpm stirrer speed was applied in DMEM FBS-free media. Data were acquired in pmol of O_2_ per second per million cells. 75 × 10^4^ ADSC were used for each experiment.

#### Mitochondrial morphology analysis

Mitochondrial morphology was performed by live-cell imaging methodology by MitoTracker™ Red CMXRos staining using EVOS® FL Auto Imaging System (AMAFD1000) microscope. Images were analyzed using the Java image processing software ImageJ FIJI version 1.54 and Mitochondria Analyzer plugin [101, 102]. The Mitochondria Analyzer is specifically designed to assess individual mitochondria morphology and complexity. We evaluated mitochondrial size (area and perimeter), shape (aspect/ratio and form factor), as well as mitochondrion network complexity (branch junctions, branches, and branch length).

### Lipid content accumulation assay

Lipid content accumulation was evaluated by AdipoRed™ (Lonza, USA). After 10 days of treatment, 2 × 10^4^ ADSC were washed with PBS 1X and incubated with the staining solution containing 7.5 μL/mL AdipoRed™ and incubated for 15 min at room temperature until image acquisition. As a positive control, ADSC were induced to differentiate into preadipocytes using adipogenesis-inducing molecules [DMEM high glucose concentration (4.5 g/L) supplemented with 10% FBS, 200 μM indomethacin, 10 μM insulin, 0.5 μM 3-isobutyl-1-methylxanthine (IBMX), 1 μM dexamethasone and 10 μM rosiglitazone] [103]. Image acquisition is described in the next section.

### Image acquisitions

Image acquisition was performed using the EVOS® FL Auto Imaging System (AMAFD1000) microscope. For each sample, 2 × 10^4^ ADSC previously seeded were treated for 10 days in 24-well culture plates. Images of three fields were acquired and the fluorescence of MitoTracker™ Green FM and MitoTracker™ Red CMXRos or AdipoRed™ was analyzed and quantified in 8-bit after transforming the images into grayscale, using the Java image processing software ImageJ version 1.8.0. Cells were analyzed at a 200 μm scale (200X magnification), randomly chosen among the fields.

### RNA extraction and cDNA synthesis

Total RNA was extracted from 1 × 10^4^ ADSC after 10 and 18 days of treatment using the TRIzol reagent (Invitrogen, USA), according to the manufacturer’s instructions. The purity of the total RNA was evaluated in a spectrophotometer by analyzing the absorbance ratio at 260/280 nm using NanoDrop Lite (Thermo Fisher, USA). Complementary DNA (cDNA) was synthesized from 1 μg of total RNA using the High-Capacity cDNA Reverse Transcription Kit (Applied Biosystems, USA) and stored at − 20 °C until use.

### Quantitative real-time polymerase chain reaction (qPCR)

The expression of the mitochondrial function-related genes was performed using the Taq DNA polymerase enzyme (Quatro G, Brazil) and SYBR green (Molecular Probes, EUA) as a fluorescent dye, normalized by the constitutive Hypoxanthine Phosphoribosyltransferase 1 (*HPRT1*) gene expression (Supplementary Table 1). The gene expression of *CDKN1A* (cyclin-dependent kinase inhibitor 21) and *CDKN2A* (cyclin-dependent kinase inhibitor 2 A) was performed using MasterMix Taqman/Probe (Quatro G, Brasil) and normalized by the constitutive gene expression of Ribosomal Protein Lateral Stalk Subunit P0 (*RPLP0*). All reactions were performed in a 96-well StepOnePlus™ instrument (Applied Biosystems, USA) using 5 μL of cDNA (1:10) as a template for qPCR reactions. All reactions included one positive and one negative control. Replicates with a standard deviation ≥ 0.3 cΤ were excluded and repeated. The data was analyzed and expressed by the 2^−ΔΔcΤ^ comparative method [104].

### Inflammatory cytokine profile

Cytokine levels were measured in the culture medium of ADSC after 0, 10, and 18 days of treatment using BD™ Cytometric Bead Array with the Human Inflammatory Kit (BD Biosciences, USA) according to the manufacturer’s instructions. The samples were acquired in the FACSCanto™ II (BD Biosciences, USA) and analyzed with the FCAP Array v3.0.1 software (Soft Flow Inc., Pecs, Hungary). Standard curves were run in duplicate, and results were expressed as picograms per milliliter (pg/mL).

### Statistics

Continuous variables from demographic data are reported as median and interquartile range (IQR), and categorical data by absolute and relative frequency. The Shapiro-Wilk test was used to test the Gaussian distribution for each data, and the difference between groups was subsequently analyzed using one-way ANOVA, followed by Tukey post-test for multiple comparisons. The Pearson test was performed to evaluate the correlation between TRF1 and C_12_FDG expression. Additional analyses were performed using the area under the curve (AUC) followed by ANOVA, to evaluate the cumulative effect of treatment in CPD and NMA. GraphPad Prism version 9.0 (LCC, California, USA) and Statistical Package for Social Sciences (SPSS) version 22 (IBM Corp. Armonk, New York, USA) were used for analysis. All tests were two-tailed, and the differences were considered when p < 0.05 (*), p < 0.01 (**), p < 0.001 (***), and p < 0.0001 (****).

### Electronic supplementary material

Below is the link to the electronic supplementary material.

Supplementary Material 1

Supplementary Material 2

Supplementary Material 3

## Data Availability

This published article and its supplementary information files include all data generated and analyzed during this study. The data that support the findings of this study are available on request from the corresponding author.
