# Supplementary material for: Obesity drives adipose-derived stem cells into a senescent and dysfunctional phenotype associated with P38MAPK/NF-KB axis
Source: Immun Ageing. 2023 Oct 11;20:51. doi: 10.1186/s12979-023-00378-0 (PMC10566105; doi:10.1186/s12979-023-00378-0)
Supplement: Supplementary file 3 — Supplementary Material 3 [file 12979_2023_378_MOESM3_ESM.pdf]

Supplementary Table 1. Primers' sequence and specific conditions.

Gene reference	Target	Primer sequences	Amp (pb)	Ann (°C)	[nM]	
					For	Rev
NM_033540.3	*MFN1*	5' AGGAACTGATGGAGATAAAGCC 3'<br>5' ACACGTACAAGACAGCCAG 3'	132	62	100	100
NM_014874.3	*MFN2*	5' TCAGGAATAAAGCCGGTTGG 3'<br>5' TGGGTGCTTCATTCTCTTGG 3'	199	60	200	200
NM_130834.3	*OPA1*	5' AATGTAATGAGGAGCACCCAG 3'<br>5' CGACAAAGGTTACAATGGTTTAGAG 3'	172	60	200	200
NM_016068.3	*FIS1*	5' TGACATCCGTAAAGGCATCG 3'<br>5' CTTCTCGTATTCCTTGAGCCG 3'	121	62	200	200
NC_012920.1	*MT-ND1*	5' GGAGTAATCCAGGTCGGT 3'<br>5' TGGGTACAATGAGGAGTAGG 3'	265	60	200	200
NM_000194.3	*HPRT1*	5' TGCTGAGGATTTGGAAAGGG 3'<br>5' ACAGAGGGCTACAATGTGATG 3'	115	60	100	100

Abbreviations: Amp (bp): Amplicon size in base pair; Ann (°C): Annealing temperature in Celsius; [nM]: Primer concentration in nanomolar; For: Forward; Rev: Reverse. *MFN1*: Mitofusin 1; *MFN2*: Mitofusin 2; *OPA1*: Optic Atrophy 1; *FIS1:* Mtochondrial Fission 1; *MT-ND1*: Mitochondrially Encoded NADH:Ubiquinone Oxidoreductase Core Subunit 1; *HPRT1*: Hypoxanthine Phosphoribosyltransferase.
